# Canadian 24-h Movement Guidelines, Life Stress, and Self-Esteem Among Adolescents

**DOI:** 10.3389/fpubh.2022.702162

**Published:** 2022-02-25

**Authors:** Hugues Sampasa-Kanyinga, Amanda Lien, Hayley A. Hamilton, Jean-Philippe Chaput

**Affiliations:** ^1^Healthy Active Living and Obesity Research Group, Children's Hospital of Eastern Ontario Research Institute, Ottawa, ON, Canada; ^2^School of Epidemiology and Public Health, University of Ottawa, Ottawa, ON, Canada; ^3^Faculty of Medicine, University of Toronto, Toronto, ON, Canada; ^4^Centre for Addiction and Mental Health, Institute for Mental Health Policy Research, Toronto, ON, Canada; ^5^Dalla Lana School of Public Health, University of Toronto, Toronto, ON, Canada

**Keywords:** physical activity, screen time, sleep, stress, self-esteem, adolescents

## Abstract

**Background:**

Adolescence is often considered a period of heightened stress, and healthy active living behaviors may help those experiencing it to better cope with life stressors and increase their self-esteem. The 24-h movement guidelines for children and adolescents recommend ≥60 min per day of moderate-to-vigorous physical activity, ≤ 2-h per day of recreational screen time, and 9–11-h of sleep per night for school-aged children or 8–10-h per night for adolescents. The objective of this study was to examine the association of meeting the 24-h movement guidelines with life stress and self-esteem among students in Ontario, Canada.

**Methods:**

Self-reported data on movement behaviors, life stress and self-esteem were derived from the 2019 cycle of the Ontario Student Drug Use and Health Survey, a cross-sectional and province-wide survey of students in grades 7–12 aged 11 to 20 years (*N* = 6,932). Multivariable ordered logistic regression analyses were adjusted for the complex sample design of the survey and for important covariates.

**Results:**

Overall, meeting all combinations of movement behavior recommendations were associated with lower life stress and better self-esteem compared with meeting none of the recommendations, except meeting the physical activity only or screen time only recommendations that were not associated with lower life stress. Meeting all 3 recommendations was associated with lower life stress (OR: 0.40; 95 CI: 0.30–0.53) and better self-esteem (OR: 0.29; 95% CI: 0.21–0.40). There was a dose-response gradient between the number of recommendations met (3 > 2 > 1) and lower life stress (*p* < 0.001) and higher self-esteem (*p* < 0.001), with meeting all 3 recommendations being the best combination.

**Conclusions:**

These findings suggest that meeting the recommendations of the 24-h movement guidelines is associated with lower life stress and better self-esteem among adolescents.

## Introduction

Adolescence is the period of transition between childhood and adulthood. It is a phase when adolescents experience rapid physical, cognitive and psychosocial growth, thus affecting how they feel, think, make decisions, and interact with the world around them (1). It is also the time when there is change in how an individual responds to stressors (2). Specifically, adolescence is marked by significant shifts in the hypothalamic-pituitary-adrenal axis reactivity, resulting in heightened stress-induced hormonal responses (3). Stress is a normal response to situational pressures or demands and is a part of everyday life, but it can have devastating effects when it becomes chronic. Self-esteem represents an overall reflection of an individual's self-worth, and encompasses beliefs about oneself as well as an emotional response to those beliefs (4). Self-esteem affects many of the developmental challenges adolescents have to deal with, such as identity formation and reshaping social relations (5, 6). Research has shown that stress and lower self-esteem increase the risk of developing mental health problems, such as depression, anxiety, suicidal ideation as well as health-compromising behaviors such as drug abuse, violent behavior, or disordered eating behaviors during adolescence (7–12). It is therefore crucial to identify factors that may enhance the ability to cope with life stressors and increase self-esteem among adolescents to inform prevention and intervention efforts.

Prior research has shown that movement behaviors, including physical activity, screen time, and sleep duration are individually associated with less stress and self-esteem. For instance, there has been consistent evidence potentiating a positive relationship between physical activity and lower stress levels and better self-esteem in adolescents (13–15), though a more recent review suggests that the long-term relationship between physical activity and self-esteem needs to be explored further as there remains a paucity of high-quality evidence (16). In terms of screen time, some relationships have been established, with higher screen time being associated with lower self-esteem and greater perceived stress in adolescents (17–21). Similarly, less stress and better self-esteem were also observed among adolescents that had longer sleep durations (22–25). For example, Fredriksen et al. (23) found that getting enough sleep prospectively predicted better self-esteem in a sample of adolescents. Although relationships between each of the individual movement behaviors and stress and self-esteem have been explored, expanding the scope of understanding to encompass the relationship between these outcomes with combinations of movement behaviors is important because physical activity, screen time, and sleep duration are codependent and can have a combined influence (26). Considering movement behaviors performed over the entire 24-h period can help inform and improve initiatives, programs, and campaigns targeted toward promoting adolescent self-esteem and alleviating stress.

The Canadian 24-h movement guidelines for children and adolescents are the first evidence-based movement guidelines that address an entire day (27). The guidelines recommend that children and adolescents have at least 60 min of moderate-to-vigorous physical activity per day involving a variety of aerobic activities. Vigorous physical activities, and muscle and bone strengthening activities should each be incorporated at least 3 days per week. Children and adolescents should also spend several hours a day engaging in a variety of structured and unstructured light physical activities. The guidelines also recommend that children and adolescents limit their recreational screen time to no more than 2-h per day, limit their sitting for extended periods, and sleep for 9 to 11-h per night (children aged 5 to 13 years) or 8- to 10-h per night (adolescents aged 14 to 17 years) (27). Meeting the guidelines has not only been positively associated with various measures of physical health such as reduced obesity and cardiometabolic risk (28), but also with measures of cognitive, social, and mental health (29–34).

From a holistic perspective, meeting the guidelines has been associated with positive global measures of health, including self-rated health and mental health (35). These global measures are shaped not only by factors of physical well-being, but also by their interplay with psychological factors and those of social functioning (36). Two such psychosocial factors that have been linked to health and health behaviors include stress and self-esteem (9–12). Despite relationships having been established between meeting the 24-h movement guidelines and health as well as between health and stress and self-esteem, the link between meeting the 24-h movement guidelines with stress and self-esteem among adolescents remains largely unknown.

Therefore, this study aims to examine the association between meeting combinations of the 24-h movement guidelines for physical activity, recreational screen time, and sleep duration with life stress and self-esteem among Ontario adolescents. We hypothesized that adherence to the 24-h movement guidelines would be associated with less life stress and better self-esteem.

## Materials and Methods

### Design

This study utilizes data from the 2019 cycle of the Ontario Student Drug Use and Health Survey (OSDUHS), a repeated cross-sectional survey that collects surveillance data related to drug use, mental health, physical health, gambling, bullying, and other risk behaviors in students in grades 7 through 12 in publicly funded schools in the province of Ontario, Canada (37). OSDUHS has been administered biennially in a random sample of Ontario schools since 1977. The survey uses a stratified two-stage cluster design (school, class) and involved students from 47 school boards, 263 schools, and 992 classrooms in the 2019 cycle. The student participation rate was 59%, which is considered above average for a student survey that required active consent from a parent or guardian (38). Non-participating students were those who were absent (12%), whose parents refused consent, or who failed to return consent forms (29%). All subjects gave their signed assent in addition to parentally signed consent for those aged under 18 years before they participated in the study. Research ethics approval for the 2019 OSDUHS was obtained from the Research Ethics Boards of the Centre for Addiction and Mental Health and York University, as well as 34 school board research review committees. The study design and methods are described in greater detail elsewhere (37).

### Participants

A total of 14,142 students aged 11 to 20 years or over completed an anonymous self-administered pen and paper questionnaire in class (37). Two survey versions (A and B) were assigned randomly to respondents. The present study was restricted to the half-sample that completed form A, which contained questions about life stress and self-esteem (*n* = 7,617). Participants were further excluded if information on any of the variables used in this study were missing. This left a final analytic sample of 6,932 students. Participants with missing data were more likely to be boys, in lower grades, and of Black ethnoracial background.

### Measures

Life stress was measured using an item that asked participants if they felt that they were under any stress, strain, or pressure in the last 4 weeks. Response options included “yes, almost more than I could take,” “yes, a lot,” “yes, some,” “yes, a little,” and “not at all.” Response options were reversed coded and treated as an ordinal variable ranging from 1 (not at all) to 5 (yes, almost more than I could take), so that higher scores indicate higher life stress. This item is used in the British Columbia Adolescent Health Survey (39); however, its psychometric properties have not been reported previously; therefore, the validity and reliability are unknown.

Self-esteem was measured using a global measure from the Rosenberg Self-Esteem Scale (40), that asked participants how much they agree with the following statement: On the whole, I am satisfied with myself. Response options included “strongly agree,” “somewhat agree,” “somewhat disagree,” and “strongly disagree.” Response options were treated as an ordinal variable ranging from 1 (strongly agree) to 4 (strongly disagree), where higher scores indicate lower self-esteem. The Rosenberg Self-Esteem Scale is the most widely used measure of self-esteem for research purposes, and it has demonstrated good reliability and validity among adolescents (41, 42).

The measures for individual movement behaviors were obtained from the Centre for Disease Control (CDC)'s Youth Risk Behavior Survey (YRBS) (43).

Physical activity was measured using an item that asked participants to indicate on how many days of the last 7 days they were physically active for a total of at least 60 min each day. Students were asked to add up all the time they spent in any kind of physical activity that increased their heart rate and made them breathe hard some of the time. Some examples were provided, such as brisk walking, running, rollerblading, biking, dancing, skateboarding, swimming, soccer, basketball, and football. Participants were also asked to include both school and non-school activities. Response options ranged from 0 to 7 days. For analysis, “7 days” corresponded to students that met the physical activity guideline recommendation (27). The remaining responses were combined to represent those who did not meet the physical activity recommendation. This self-reported measure has shown good validity in comparison with accelerometry measures among children and adolescents (44).

Screen time was assessed using an item that asked students to indicate how many hours a day, on average, they spend watching TV/movies/videos, playing video games, texting, messaging, posting, or surfing the Internet in their free time in the last 7 days. This includes time on any screen, such as a smartphone, tablet, TV, gaming device, computer, or wearable technology. Participants who spent no more than 2-h per day of screen time were considered meeting the screen time recommendation (27). Those who exceeded 2-h per day were considered not meeting the screen time recommendation. Self-reported measures of recreational screen time have demonstrated good psychometric properties among children and adolescents (45, 46).

Sleep duration was assessed using an item that asked participants to indicate how many hours of sleep they get on an average school night. Students who reported a sleep duration within the recommended range (9–11 h per night for 11–13-year-olds; 8–10 h per night for 14–17-year-olds, or 7–9 h per night for those aged 18 years or older) were considered meeting the sleep duration recommendation. In contrast, those who reported sleep duration below the range for their age group were considered not meeting the sleep duration recommendation (27, 47). This self-reported measure of sleep duration has shown good validity and reliability among children and adolescents (48).

Covariates included within analyses were age, gender (boys or girls), ethnoracial background (White, Black, East/Southeast Asian, South Asian, Latino, or other), subjective socioeconomic status, and body mass index (BMI) z-scores. Subjective SES was assessed using an adapted version of the MacArthur Scale of Subjective Social Status (49, 50). The MacArthur Scale has been shown to be a reliable measure of subjective social status (50). Students self-reported their body weight (kilograms) and height (meters). BMI (kilograms/meters^2^) was calculated and converted into z-scores following the World Health Organization reference data (51).

### Data Analysis

All analyses applied sample weights and adjusted for the complex survey design within Stata 16.0 (Stata Corporation, College Station, TX, USA). Proportions and means were used to describe the data. Gender differences across characteristics were assessed using the design-adjusted Rao-Scott F-test statistic for categorical variables and an adjusted Wald test for continuous variables. Two-way interactions were used to investigate whether the associations of meeting the 24-h movement guidelines with life stress and self-esteem varied by gender. Given that gender was not a significant moderator of the association of meeting the 24-h movement guidelines with life stress or self-esteem, data were pooled to maximize sample size. Separate univariable and multivariable ordered logistic regression analyses were performed to examine the association of meeting the 24-h movement guidelines with the outcome variables of life stress and self-esteem. Multivariable models were adjusted for age, gender, ethnoracial background, subjective socioeconomic status, and BMI z-score. Age, subjective SES, and BMI z-scores were treated as continuous variables, whereas gender and ethnoracial background were treated as categorical variables. Both unadjusted and adjusted odds ratio (OR) and their 95% confidence intervals (CI) are presented. The strength of the association (as measured by the OR) and CIs for meeting each guideline was used to determine which behavior has the strongest relationship with life stress and self-esteem. Finally, life stress was included in separate models for self-esteem to investigate the interrelation between life stress and self-esteem before (models partially adjusted) and after adjusting for other covariates (models fully adjusted). This is particularly important because research has shown that self-esteem affects both the way individuals react to a stressful event and cope with it (52). Conversely, a stressful event can negatively affect self-esteem (52). In order to determine the strongest predictor of high life stress and low self-esteem, we conducted a one-way ANOVA for each outcome and used Tukey *post hoc* tests to identify which movement behaviors were significantly different from one another.

## Results

The sample characteristics are outlined in Table 1. More than half of the sample was girls, and 51% reported that they were of White ethnoracial background. Overall, 19.8% of participants met the physical activity recommendation, 24.3% met the screen time recommendation, and 34.2% met the sleep duration recommendation. A total of 44.5% met none of the recommendations, whereas 3.5% of participants met all 3 recommendations. Boys were more likely than girls to meet the physical activity and sleep duration recommendations. They were also more likely to meet all 3 recommendations than girls (*p* < 0.001). Girls were more likely than boys to report higher levels of life stress (*p* < 0.001) and lower self-esteem (*p* < 0.001).

**Table 1:** Descriptive characteristics of participants.

	**Full sample (*n* = 6,932)**	**Boys (*n* = 2,997)**	**Girls (*n* = 3,935)**
**Age (years)**			
Mean (SD)	15.2 (1.8)	15.2 (1.6)	15.2 (1.9)
**Ethnic background**			
White	50.9	49.9	52.0
Black	9.4	9.3	9.5
East/South-East Asian	14.0	15.3	12.6
South Asian	9.0	9.2	8.7
Other	16.7	16.3	17.1
**Subjective socioeconomic status**			
Mean (SD)	6.9 (1.7)	7.0 (1.6)	6.9 (1.8)
**Body mass index z-score**			
Mean (SD)	0.3 (1.1)	0.4 (1.0)	0.3 (1.3)
Physical activity^***^			
Not meeting	80.2	74.7	85.9
Meeting	19.8	25.3	14.1
**Screen time**			
Not meeting	75.7	75.0	76.5
Meeting	24.3	25.0	23.5
Sleep duration^***^			
Not meeting	65.8	61.4	70.4
Meeting	34.2	38.6	29.6
Combinations of movement recommendations^***^			
Meeting none	44.5	39.3	49.8
Physical activity only	8.0	9.3	6.5
Screen time only	9.8	8.0	11.6
Sleep duration only	18.5	19.8	17.1
Physical activity and screen time only	3.6	4.7	2.5
Physical activity and sleep only	4.7	6.4	3.0
Screen time and sleep duration only	7.4	7.5	7.4
Meeting all 3 recommendations	3.5	4.8	2.1
Life stress^***^			
Not at all	14.3	19.9	8.5
A little	27.1	31.4	22.8
Some	25.3	24.8	25.9
A lot	22.1	16.4	27.9
Almost more than I could take	11.2	7.4	15.0
Self-esteem (satisfied with myself)^***^			
Strongly agree	29.1	37.8	20.1
Somewhat agree	43.6	43.0	44.3
Somewhat disagree	18.0	13.5	22.6
Strongly disagree	9.3	5.7	12.9

*Data are shown as column %, unless otherwise indicated. SD, standard deviation*.

*** * p of differences between boys and girls <0.001*.

Results of the univariable and multivariable ordered logistic regression models examining the association of meeting the 24-h movement guidelines with life stress and self-esteem are presented in Table 2. After adjusting for age, gender, ethnoracial background, subjective socioeconomic status and BMI z-score, results indicate that meeting all 3 recommendations was associated with lower life stress (OR: 0.40; 95% CI: 0.30–0.53; *p* < 0.001) and better self-esteem (OR: 0.29; 95% CI: 0.21–0.40; *p* < 0.001). Other combinations of movement behavior recommendations were also associated with lower life stress and better self-esteem, except meeting the physical activity only and screen time only recommendations, which were not associated with life stress. Results from one-way ANOVA and *post-hoc* comparisons between estimates of physical activity, screen time, and sleep duration suggest that sleep duration is the strongest correlate of high life stress compared physical activity (*t* = −3.29; *p* = 0.022) or screen time (*t* = −5.55; *p* < 0.001). However, for lower self-esteem, sleep duration was more strongly associated with this outcome than screen-time (*t* = −4.13; *p* = 0.001), but not physical activity (*t* = −1.83; *p* = 0.596). There were no significant differences between sleep duration and physical activity in their associations with self-esteem.

**Table 2:** Results of the ordered logistic regression analysis of the associations of meeting different combinations of movement behavior recommendations with life stress and self-esteem among adolescents (*N* = 6,932).

	**Higher life stress**		**Lower self-esteem**	
	**OR**	**95% CI**	**OR**	**95% CI**
**Unadjusted**				
Meeting none	1		1	
Physical activity only	0.78^*^	0.61–0.99	0.57^***^	0.45–0.73
Screen time only	0.94	0.79–1.11	0.79^*^	0.62–0.99
Sleep duration only	0.51^***^	0.43–0.61	0.53^***^	0.46–0.62
Physical activity and screen time only	0.44^***^	0.32–0.61	0.38^***^	0.27–0.53
Physical activity and sleep only	0.30^***^	0.22–0.40	0.28^***^	0.21–0.36
Screen time and sleep duration only	0.33^***^	0.27–0.41	0.23^***^	0.18–0.30
Meeting all 3 recommendations	0.25^***^	0.19–0.32	0.17^***^	0.13–0.23
**Adjusted**				
Meeting none	1		1	
Physical activity only	1.01	0.81–1.28	0.72^**^	0.57–0.90
Screen time only	0.95	0.81–1.12	0.81^*^	0.66–0.99
Sleep duration only	0.57^***^	0.48–0.67	0.62^***^	0.53–0.72
Physical activity and screen time only	0.60^**^	0.42–0.86	0.51^***^	0.37–0.72
Physical activity and sleep only	0.44^***^	0.33–0.59	0.41^***^	0.32–0.53
Screen time and sleep duration only	0.41^***^	0.33–0.51	0.29^***^	0.22–0.37
Meeting all 3 recommendations	0.40^***^	0.30–0.53	0.29^***^	0.21–0.40

*OR, odds ratio; CI, confidence interval*.

*Covariates in the adjusted models included age, gender, ethnoracial background, subjective socioeconomic status, and body mass index z-scores*.

* *p <0.05*;

** *p <0.01*;

*** *p <0.001*.

Table 3 outlines results of the univariable and multivariable ordered logistic regression models examining the association of number of movement behavior recommendations met with life stress and self-esteem. There was a dose-response gradient between the number of recommendations met (3 > 2 > 1) and lower life stress (*p* < 0.001) and better self-esteem (*p* < 0.001), with meeting all 3 recommendations being the best combination. Gender was not a significant moderator of these relationships.

**Table 3:** Results of the ordered logistic regression analysis of the associations of number of movement behavior recommendations met with life stress and self-esteem among adolescents (*N* = 6,932).

	**Higher life stress**		**Lower self-esteem**	
	**OR**	**95% CI**	**OR**	**95% CI**
**Unadjusted**				
None	1		1	
One	0.66^***^	0.58–0.75	0.60^***^	0.53–0.69
Two	0.35^***^	0.29–0.41	0.28^***^	0.23–0.33
Three	0.25^***^	0.19–0.32	0.17^***^	0.13–0.23
*p-*trend^***^				
**Adjusted**				
None	1		1	
One	0.74^***^	0.65–0.84	0.69^***^	0.61–0.78
Two	0.46^***^	0.38–0.54	0.37^***^	0.31–0.44
Three	0.40^***^	0.31–0.53	0.29^***^	0.21–0.40
*p-*trend^***^				

*OR, odds ratio; CI, confidence interval*.

*Covariates in the adjusted models included age, gender, ethnoracial background, subjective socioeconomic status, and body mass index z-scores*.

*** *p <0.001*.

Results of analyses including life stress and self-esteem in the same models are summarized in Table 4. The association between meeting different combinations of movement behavior recommendations and self-esteem was independent of life stress and other important covariates. Meeting all 3 recommendations (OR: 0.35; 95% CI: 0.25–0.49; *p* < 0.001) or different combinations of movement behavior recommendations were associated with better self-esteem. Findings also indicate that high life stress is a significant correlate of low self-esteem before and after adjusting for other important covariates (OR: 1.62; 95% CI: 1.52–1.71; *p* < 0.001).

**Table 4:** Results of the ordered logistic regression analysis of the association of meeting different combinations of movement behavior recommendations with self-esteem independent of life stress among adolescents (*N* = 6,932).

	**Model 1**		**Model 2**	
	**OR**	**95% CI**	**OR**	**95% CI**
Higher life stress	1.75^***^	1.65–1.86	1.62^***^	1.52–1.71
**Combination of movement behaviors**				
Meeting none	1		1	
Physical activity only	0.61^***^	0.48–0.76	0.70^**^	0.56–0.88
Screen time only	0.79^*^	0.63–0.98	0.80^*^	0.66–0.98
Sleep duration only	0.66^***^	0.56–0.77	0.72^***^	0.62–0.85
Physical activity and screen time only	0.49^***^	0.34–0.72	0.60^**^	0.42–0.85
Physical activity and sleep only	0.39^***^	0.29–0.51	0.49^***^	0.38–0.64
Screen time and sleep duration only	0.31^***^	0.24–0.40	0.35^***^	0.27–0.46
Meeting all 3 recommendations	0.25^***^	0.18–0.34	0.35^***^	0.25–0.49

*OR, odds ratio; CI, confidence interval*.

*Model 1 is partially adjusted (i.e., it includes higher life stress only). Model 2 is fully adjusted (i.e., it includes higher life stress in addition to other covariates, including age, gender, ethnoracial background, subjective socioeconomic status, and body mass index z-scores*.

* *p <0.05*;

** *p <0.01*;

*** *p <0.001*.

## Discussion

The objective of this study was to examine the association between meeting combinations of the 24-h movement behavior recommendations for physical activity, recreational screen time, and sleep duration with life stress and self-esteem in a representative and province-wide sample of Ontario adolescents. Consistent with our hypothesis, we found that adherence to the 24-h movement guidelines is associated with less life stress and better self-esteem. Intermediate combinations of movement behavior recommendations were also associated with lower life stress and better self-esteem, with a few exceptions. Moreover, there was a dose-response gradient between the number of recommendations met and lower life stress and better self-esteem, in that adherence to a greater number of recommendations was more strongly associated with less life stress and better self-esteem among adolescents. Although girls were more likely than boys to report higher levels of life stress and lower self-esteem, gender was not a significant moderator of the associations of meeting the 24-h movement guidelines with life stress or self-esteem, suggesting that the associations did not differ between boys and girls. Results further indicated that sleep duration had the strongest association with life stress and self-esteem among the three movement behaviors. The only exception was the association with self-esteem where sleep duration and physical activity were similar correlates.

Many studies have investigated the relationships between physical activity, recreational screen time, and sleep duration with life stress and self-esteem among adolescents (13–15, 17–25), though most considered the movement behaviors individually and in isolation of each other, ignoring the intrinsic and empirical interactions between these behaviors. To our knowledge, we are the first to investigate these associations in the context of the 24-h movement guidelines (27) at the population level of adolescents. Our findings highlight the importance of being active, limiting recreational screen time, and getting enough sleep as possible strategies to better cope with life stressors and increase self-esteem. These novel findings support the need to promote adherence to the 24-h movement guidelines in children and adolescents as a possible means for promoting less life stress and better self-esteem.

Several mechanisms could explain the associations of regular physical activity, low screen time, and adequate sleep duration with low life stress and better self-esteem. Regular physical activity is thought to be associated with stress reduction and better mood (13–15). It reduces levels of the body's stress hormones, such as adrenaline and cortisol (53). It also stimulates the production of endorphins, which are chemicals in the brain that are the body's natural painkillers and mood elevators (54). Regular physical activity is also associated with better self-esteem, because sometimes self-esteem issues among adolescents are tied to body perception (55, 56). Regular physical activity helps build confidence by improving body image, and thus contributes to a better self-esteem (15). Physical activity also generates a sense of accomplishment that boosts confidence and puts one's mind in a more positive state.

Excessive recreational screen time is known to be associated with a wide range of negative outcomes, including but not limited to physical (e.g., excess body weight, cardiometabolic problems), social (e.g., aggressive behaviors and cyberbullying victimization), and mental health (e.g., depressive symptoms and suicidality) problems (32, 57–59). These negative outcomes could certainly constitute an important source of stress. However, it is also possible that high stress levels lead to excessive electronic media use as a possible coping strategy (60). The mechanism explaining the link between recreational screen time and self-esteem could be explained by a dissatisfaction with body weight (55, 56). Indeed, recreational screen time may expose users to unrealistic and idealized body types that are based on the stereotype of a lean body for girls and a muscular body for boys. Although perceived body image does not always reflect reality (61, 62), having a negative body image is usually related not only to a lower self-esteem, but also to unhealthy eating and weight control behaviors (63, 64), which could be an important source of stress as well.

Short sleep duration can lead to a dysregulation of the hypothalamic-pituitary-adrenal axis involving changes in cortisol secretion (3), which, in turn, increases stress (23) and affects self-esteem (65). Short sleep duration can also drive higher stress and lower self-esteem via daytime sleepiness and fatigue, which in turn could lead to difficulty concentrating or learning, increased risk of academic failure, and impaired mood (66). Another mechanism that could explain the association of short sleep duration with high stress and low self-esteem could be dissatisfaction with body weight. Research has shown that short sleep duration is associated with dieting and unhealthy weight-control behaviors among adolescents (67). Finally, short sleep duration can lead to high stress and lower self-esteem via increased depressive symptoms and anxiety (25). Inversely, research has shown that experiencing higher stress enhances the risk of sleep problems. It is also possible that less stress and better self-esteem facilitate better sleep by their beneficial influence on physical and mental well-being (68). The findings that sleep duration was the most strongly associated with life stress and self-esteem among the three movement behaviors are consistent with previous findings indicating that meeting the sleep duration recommendation has generally more incremental benefits to adolescent mental health indicators than the remaining movement behaviors (33, 69, 70).

Our results indicated that sleep duration had the strongest association with life stress and self-esteem among the three movement behaviors. The only exception was the association with self-esteem where sleep duration and physical activity were similar correlates. These findings are somewhat consistent with those from previous studies suggesting that sleep duration may be the most important correlate of mental health outcomes compared to screen time and physical activity (33, 34, 70). For example, Sampasa-Kanyinga et al. (69) found that meeting the sleep duration recommendations was the strongest predictor of depressive and anxiety symptoms using the 2017 cycle of the OSDUHS. Similarly, Patte et al. (71) found that meeting the sleep duration recommendations was more strongly associated with depressive symptoms in a longitudinal sample of Canadian adolescents. Nevertheless, meeting the physical activity and screen time recommendations should not be neglected because of their proven multiple health benefits among children and youth (21, 72). These findings underscore the need to promote adherence to the 24-h movement guidelines, particularly sleep duration among adolescents as a possible means to protect their mental health. The finding that there are no significant differences between sleep duration and physical activity in their association with low self-esteem is interesting and deserves further investigation. It is possible that physical activity is as important as sleep duration in relation to self-esteem. In their systematic review and meta-analysis, Liu et al. (73) provided evidence that physical activity interventions are associated with increased self-esteem in children and adolescents. While it is difficult to explain why sleep duration is the most important correlate of mental health indicators among adolescents and why there were no differences between sleep duration and physical activity in their association with low self-esteem in the present study, it is possible that some methodological factors, such as the use of self-reported or single item measures of movement behaviors explain these findings. Future studies using objective measures of sleep duration and physical activity in a large and representative sample of adolescents are needed to replicate these findings.

Several limitations of this study should be noted. First, given the cross-sectional nature of the data, our findings can only provide information about associations among variables rather than indicating causal relationships. Therefore, reserve causation among the observed associations is always a possibility. For example, it is possible that adolescents meet the physical activity recommendation because they have high stress (53). Second, the data are based entirely on self-reports and may be subject to recall errors and desirability bias. For example, differential reporting of movement behaviors may have occurred if participants tended to systematically overestimate physical activity and/or sleep duration or underestimate screen time due to social desirability. Despite these limitations of self-reported measures of movement behaviors, they have been indicated to have good validity in comparison with objective measures among children and adolescents (44–46, 48). Third, this study includes students in grade 7 to 12 within the regular school system in Ontario and is not representative of all students in Canada. Fourth, the present study did not examine other components of the 24-h movement behaviors, such as light and vigorous physical activity, muscle and bone strengthening activities, and prolonged sitting. Future research capturing these indicators is needed to replicate our analyses. Finally, residual confounding by unmeasured factors (e.g., medication use, chronic conditions, family involvement) is always a possibility in epidemiology.

Despite limitations, this study provides evidence that meeting the recommendations of the 24-h movement guidelines is associated with lower life stress and better self-esteem among adolescents. These results highlight the importance of meeting the 24-h movement guidelines, and public health interventions are needed to encourage adolescents to meet these guidelines. All stakeholders that interact with youth, including health service providers, educators, schools, and parents and adolescents themselves should be aware of these findings. Future studies using a longitudinal design are needed to replicate these findings and clarify the nature of the causal pathways involved.

## Data Availability Statement

The datasets presented in this article are not readily available because of the Centre for Addiction and Mental Health's and the Ontario Public and Catholic School Board's institutional Research Ethics Board agreements. Requests to access the datasets should be directed to the Centre for Addiction and Mental Health at info@camh.ca.

## Ethics Statement

The studies involving human participants were reviewed and approved by Research Ethics Boards of the Centre for Addiction and Mental Health and York University, as well as 34 school board research review committees. Written informed consent to participate in this study was provided by the participants' legal guardian/next of kin.

## Author Contributions

HS-K, AL, HH, and J-PC participated in the conception of the study. HS-K conducted statistical analyses. HS-K and AL wrote the first version of the manuscript. HH and J-PC substantially contributed to the methods and interpretation of results. AL, HH, and J-PC critically reviewed the manuscript. All authors read and approved the final manuscript.

## Conflict of Interest

The authors declare that the research was conducted in the absence of any commercial or financial relationships that could be construed as a potential conflict of interest.

## Publisher's Note

All claims expressed in this article are solely those of the authors and do not necessarily represent those of their affiliated organizations, or those of the publisher, the editors and the reviewers. Any product that may be evaluated in this article, or claim that may be made by its manufacturer, is not guaranteed or endorsed by the publisher.
